# Safe Surgical Dislocation and Open Reduction, Internal Fixation with Herbert Screw for Pipkin's Fracture Head of Femur in an Obese Patient: An Interesting Case Report

**DOI:** 10.7759/cureus.32171

**Published:** 2022-12-03

**Authors:** Kishore Vellingiri, Hariprasad Seenappa

**Affiliations:** 1 Orthopedics, Sri Devaraj Urs Academy of Higher Education and Research, Kolar, IND

**Keywords:** herbert screw, open reduction and internal fixation, safe surgical dislocation of hip, pipkin’s fracture type 2, obese

## Abstract

Head of femur fractures are relatively rare and tend to be associated with dislocations of the hip and fractures of the acetabulum. Other parts of the femur, namely the neck is also often involved. Only two cases per million are reported on a yearly basis which poses significance due to its extreme rarity. Here, we present a 30-year-old obese male patient with Pipkin’s fracture who was treated successfully by us without any major complications.

## Introduction

Head of femur fractures is often associated with fractures of other parts of the femur, namely the neck as well as dislocations of the hip and fracture of the acetabulum [1]. Nearly two cases per million are reported on a yearly basis which poses significance due to its extreme rarity [2]. To ensure a safe and effective management of such cases, Ganz et al. proposed the "safe surgical hip dislocation" method to allow for exposure of the acetabulum and femoral head while ensuring that the blood supply to the femoral head is uninterrupted [3]. Although multiple methods have been proposed, exposure of the head of femur in such cases seems inadequate. This might be viewed as a limitation to the application of other surgical approaches to fixation of such fractures [4]. Here we present a 30-year-old obese male patient with Pipkin’s fracture who was treated successfully by us without any major complications.

This case study was previously presented as an e-poster at the "Annual Conference Delhi Orthopaedic Association" held between November 28, 2020, and November 29, 2020. The abstract of this article was published in the online supplement of the conference journal.

## Case presentation

**Figure 1:**
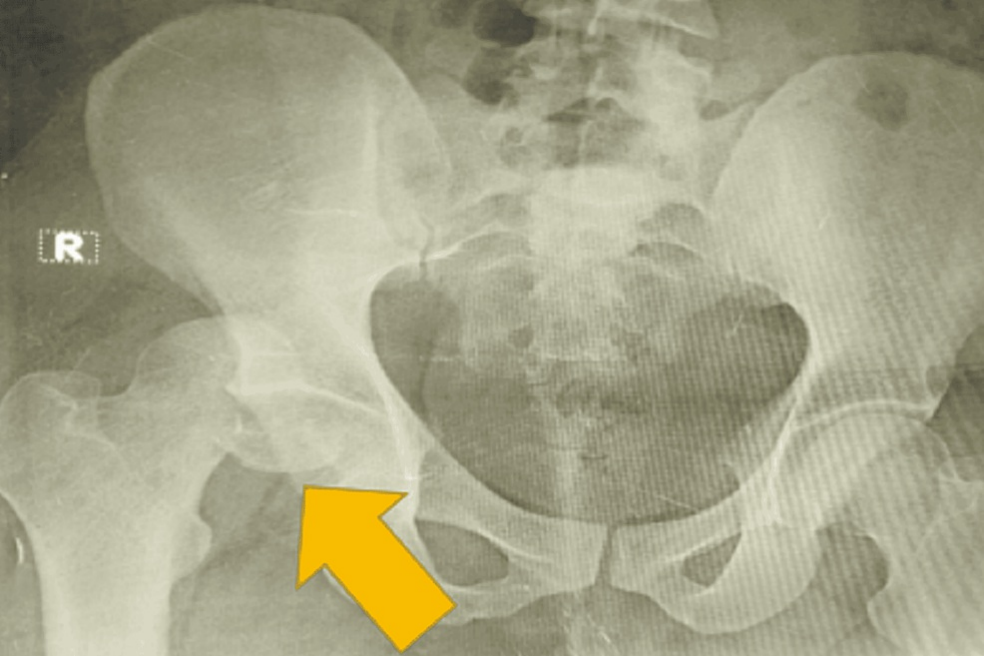
Plain radiograph of right hip showed fracture of head of right femur with posterior dislocation of right hip.

A 30-year-old class 2 obese (BMI: 35.4) male patient presented with an alleged history of road traffic accident sustaining injury to his right hip. The patient was unable to bear weight over his right lower limb since the injury. Warmth and tenderness were felt over the right hip with limb shortening on the right side. A swelling was also palpable over the right hip. Flexion, adduction, and internal rotation of the right lower limb were seen with a history of decreased range of movements. Range of movements could not be elicited in the patient as a result of pain. However, the range of motion at the ankle joint was full. Active toe movements were present. No distal neurovascular deficits were noticed. Plain radiograph of the right hip showed fracture of head of right femur with posterior dislocation of right hip (Figure 1).

**Figure 2:**
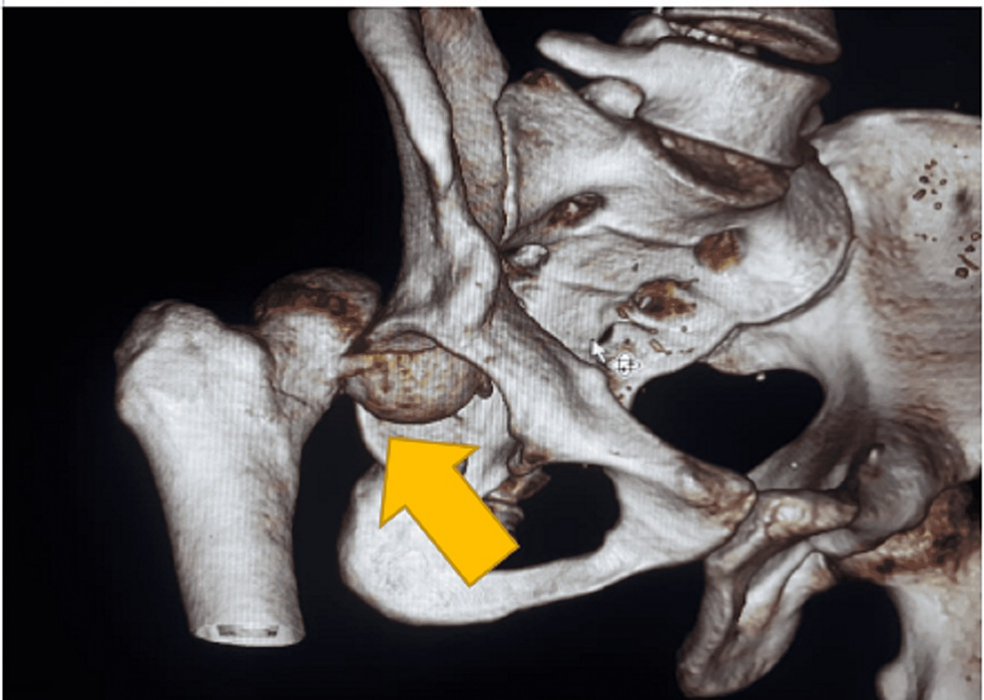
Three-dimensional reconstruction CT image showing posterior dislocation with Pipkin’s type II fracture.

**Figure 3:**
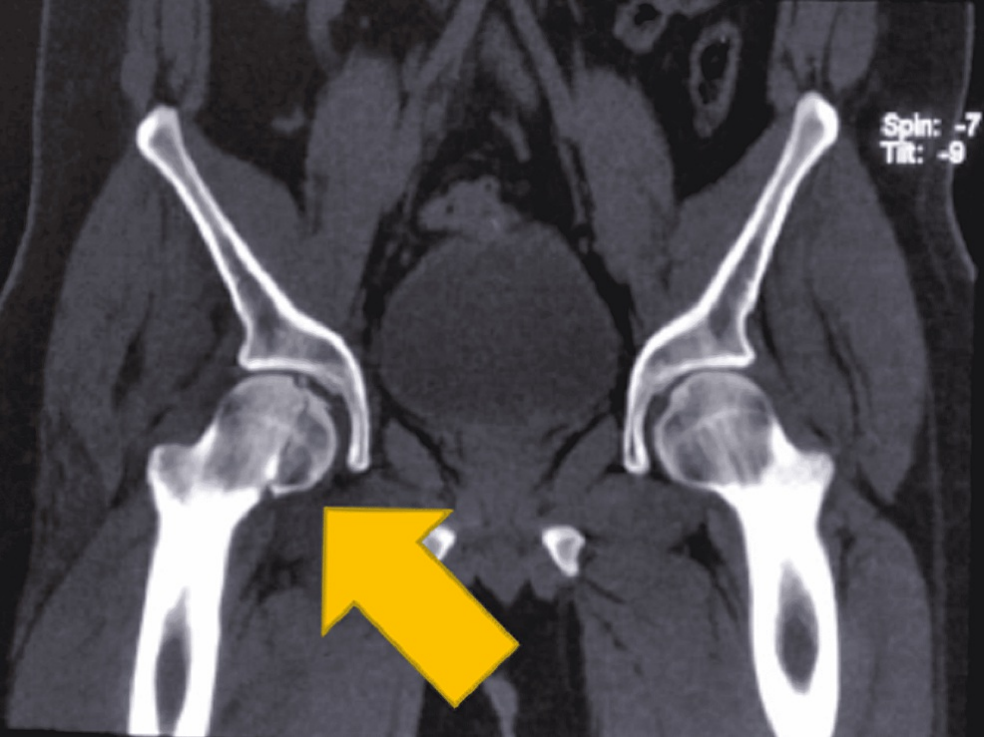
Coronal CT view showing Pipkin’s type II fracture.

Computed tomography images are shown in Figures 2, 3. After obtaining proper written, informed consent and under general anesthesia the posterior dislocation of right hip was reduced. This was confirmed using fluoroscopy. The patient was put in upper tibial skeletal traction for one week. This was followed by safe surgical dislocation with open reduction and internal fixation.

**Figure 4:**
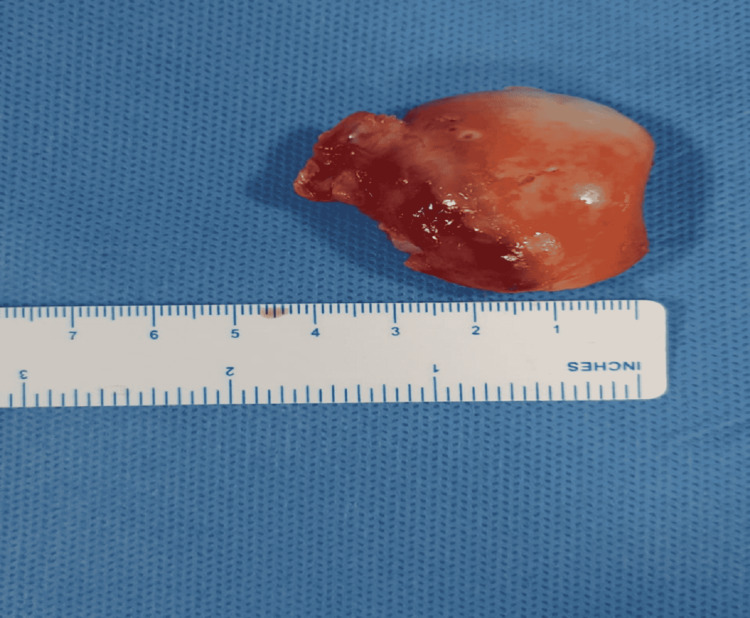
Femur head fracture fragment.

**Figure 5:**
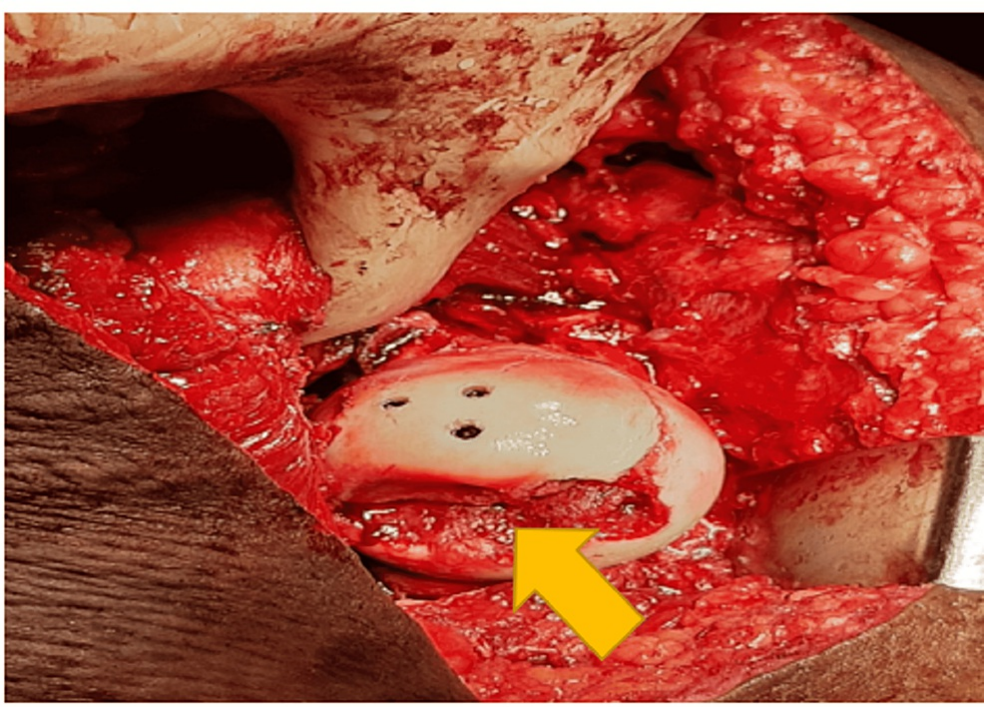
Intra-operative image of safe surgical dislocation with Pipkin’s fracture reduced with headless cancellous screw fixation.

**Figure 6:**
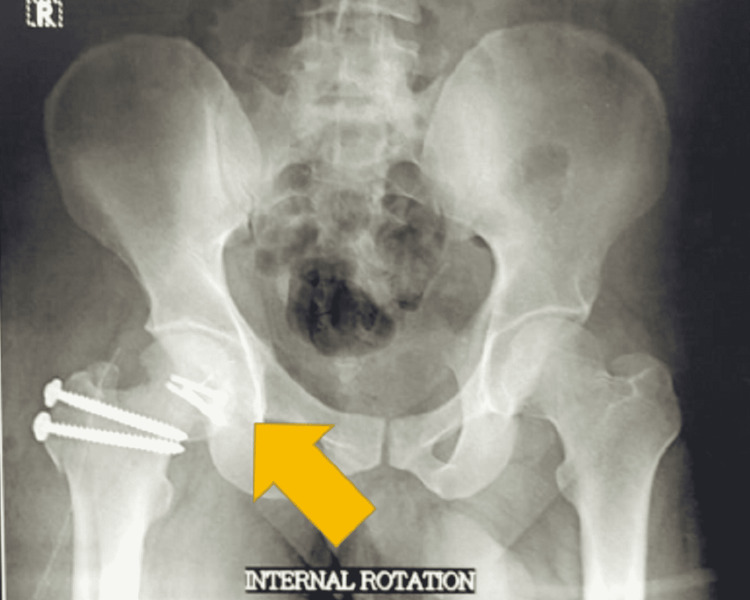
Internal rotation radiograph of pelvis anteroposterior view showing reduced Pipkin’s fracture at six months follow-up.

**Figure 7:**
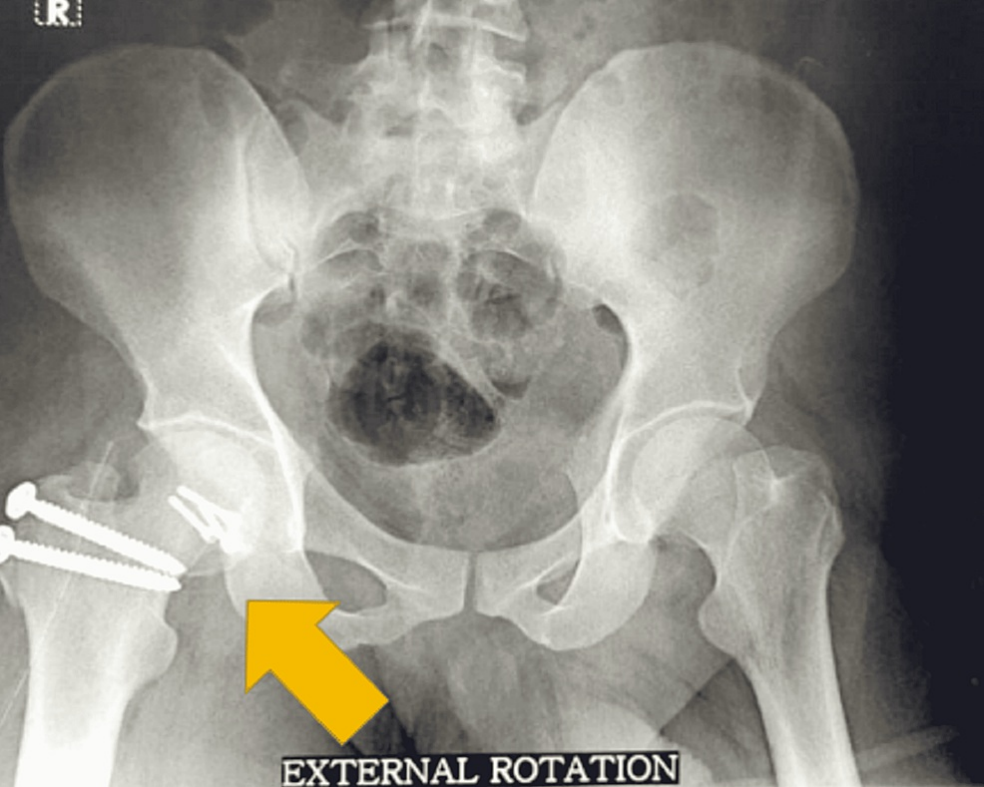
External rotation radiograph of pelvis anteroposterior view showing reduced Pipkin’s fracture at six months follow-up.

The head of femur fracture fragment is shown in Figure 4. The fixation was done using Herbert screw/Headless cancellous screw for Pipkin’s type II fracture and cancellous screw over greater trochanter of right hip as shown in Figure 5. Post-operative period was uneventful. Wound inspection was done on the second operative day. Sutures were removed on the 12th post-operative day. Physiotherapy was started on the 15th-day post-surgery. The patient was made to walk with walker assistance without weight bearing. At the time of discharge, a healed surgical scar of 20 cm was noticed over the lateral aspect of the right thigh. Range of flexion at right hip was 90° and right knee was 120°. Active ankle and toe movements were present. No distal neurovascular deficits were noticed. The patient was followed at regular intervals. Radiographs during the follow-up at six months are shown in Figures 6, 7.

## Discussion

The surgical procedure and approach used depend on the type of fracture and the associated injuries that are present in the individual. The goal of surgery is to ensure anatomical reduction of the fragments while ensuring that the injury to nearby soft tissues is minimized [5]. Precision is essential to avoid serious complications that might arise following fractures of the head of femur. Imaging should be performed in doubtful cases to avoid injury to the neck of the femur which may give rise to avascular necrosis [6].

Iatrogenic Pipkin's type III fractures can also occur as a result of forced closed reduction of femoral head fracture and dislocation injuries which is otherwise irreducible. CT and radiography can be of particular use to decide upon the safest management approach [7]. While better range of movements and recovery can be better with fragment excision, open reduction and internal fixation (ORIF) can result in an increase in the incidence of avascular necrosis. Similarly, conservative management strategies have an increased possibility for the development of arthritis. These outcomes should be kept in mind prior to making an informed decision on the management strategy of a fractured head of femur [8]. Concentric reduction of the fracture associated with early diagnosis and management is imperative to ensure maximum recovery and healing [9]. Use of bioabsorbable screws and reconstruction plates in Pipkin's type IV fractures and close reduction within six hours in the case of hip dislocation in Pipkin's type I and II fractures of the head of femur have potential for good surgical outcomes and improved joint function and range of movements. Both Smith-Peterson (S-P) and the modified Hardinge approach are effective modalities for the treatment of Pipkin's type I and II fractures of the head. Modified Hardinge is often considered superior possibly owing to decreased blood loss and shorter duration of surgery. Nevertheless, surgical outcome is described as not being linearly dependent on the specific approach tailored to the patient [10-12]. Alternative reliable methods such as trochanteric flip osteotomy can also be used in Pipkin's I and II fractures of head of femur [13]. However, despite the surgical approach tailored, case reports show similar outcomes of avascular necrosis (AVN) and increased risk of heterotopic ossification, although a significant decrease was seen in the rate of posttraumatic arthritis [14].

## Conclusions

Obtaining a better functional and clinical outcome using safe surgical dislocation and open reduction with headless cancellous screw fixation in obese patients presenting with Pipkin’s fracture type II is definitely a challenge. Further studies with larger sample size are needed to validate our study findings.
